# Development and process evaluation of a web-based responsible beverage service training program

**DOI:** 10.1186/1747-597X-7-41

**Published:** 2012-09-22

**Authors:** Brian G Danaher, Jack Dresser, Tracy Shaw, Herbert H Severson, Milagra S Tyler, Elisabeth D Maxwell, Steve M Christiansen

**Affiliations:** 1 Oregon Research Institute, 1715 Franklin Blvd, Eugene 97403, OR, USA; 2 Deschutes Research, Inc, 261 East 12th Avenue Suite 210, Eugene 97401, OR, USA; 3 InterVision Media, 261 East 12th Avenue, Suite 100, Eugene 97401, OR, USA

**Keywords:** Internet, Web program development, Responsible beverage service, Alcohol, Training

## Abstract

**Background:**

Responsible beverage service (RBS) training designed to improve the appropriate service of alcohol in commercial establishments is typically delivered in workshops. Recently, Web-based RBS training programs have emerged. This report describes the formative development and subsequent design of an innovative Web-delivered RBS program, and evaluation of the impact of the program on servers’ knowledge, attitudes, and self-efficacy.

**Methods:**

Formative procedures using focus groups and usability testing were used to develop a Web-based RBS training program. Professional alcohol servers (N = 112) who worked as servers and/or mangers in alcohol service settings were recruited to participate. A pre-post assessment design was used to assess changes associated with using the program.

**Results:**

Participants who used the program showed significant improvements in their RBS knowledge, attitudes, and self-efficacy.

**Conclusions:**

Although the current study did not directly observe and determine impact of the intervention on server behaviors, it demonstrated that the development process incorporating input from a multidisciplinary team in conjunction with feedback from end-users resulted in creation of a Web-based RBS program that was well-received by servers and that changed relevant knowledge, attitudes, and self-efficacy. The results also help to establish a needed evidence base in support of the use of online RBS training, which has been afforded little research attention.

## Background

Drunk driving crashes are responsible for nearly 11,000 fatalities and one-third of all car accident fatalities in the United States each year [1]. Approximately half of automobile drivers found to be legally impaired had consumed alcohol at licensed alcohol outlets [2]. Moreover, Naimi and colleagues [3] found that over half of binge drinkers drinking in bars, clubs and restaurants participated in drunk driving and that they consumed an average of 8.1 drinks and over 25% of them consumed more than 10 drinks. The extent of this important public health problem might therefore be reduced were licensed alcohol servers to more effectively manage and curtail the sale of alcohol to underage patrons (e.g., via ID check) and those patrons who are intoxicated or alcohol-impaired. Improved alcohol server behaviors can be addressed by a combination of community campaigns, legislation, and enforcement aimed at the consumer, and improved responsible beverage service (RBS) training of servers in commercial alcohol establishments. RBS describes the set of behavioral strategies that can be used by beverage servers to support responsible and moderate alcohol use as a way to reduce the chances that their patrons become intoxicated. RBS trainings can combine serving practices along with changes in policies for both servers and their managers [4-7].

As of 2011, 17 states in the U.S. had enacted mandatory RBS laws that required training and certification of RBS. A total of 15 of these states – including Oregon – mandate RBS training for managers and 13 states require RBS training for servers [8]. RBS certification is typically required for new servers and periodic (often annual) recertification is required. Another 19 states have voluntary laws that promote RBS training through incentives such as penalty abatement. RBS training has typically been delivered by in-person trainings or workshops by state-approved training services. An increasing number of states have supported the use of Web-based RBS training as an alternative to live trainings. As a result, Web-based RBS training programs have become more widely available.

This report describes the formative development, design, and preliminary test of a Web-based RBS training program designed to encourage responsible alcoholic beverage service by servers and managers of commercial alcohol service establishments.

## Methods

### Formative development process and program design

#### Interdisciplinary team

**Figure 1:**
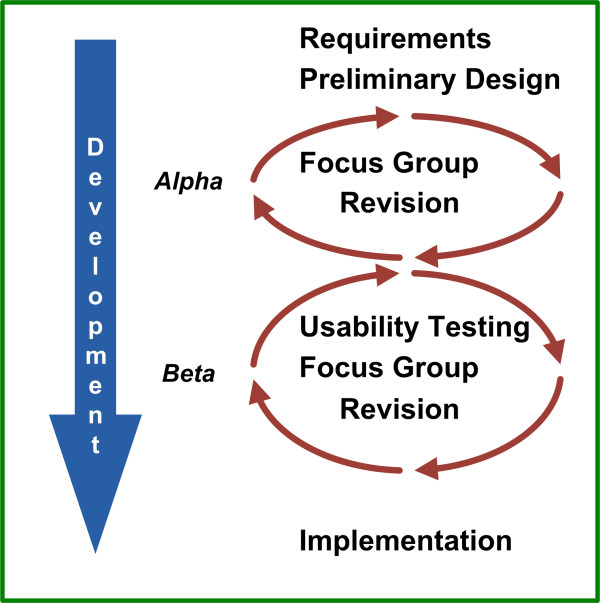
Iterative and Incremental Development Process.

Initial project development meetings were convened for a multidisciplinary team composed of individuals from Deschutes Research, Inc. (a company focused on the dissemination of evidence-based programs), InterVision Media (a Oregon-based technology media and Web intervention development programs), and Oregon Research Institute (a non-profit independent behavioral sciences research center) to generate ideas regarding the design, development, and evaluation of a Web-based RBS training program. Team members had experience and expertise in the development and evaluation of alcohol treatment programs, health behavior change programs, Web-based behavioral interventions, and the programming of complex interactive websites. In addition, the team had been previously involved in a Phase I SBIR research program that developed and tested a portion of the full RBS Web-based program that is described in this report. The team followed an incremental and iterative formative development process [9] spanning the steps between initial design and eventual program testing (see Figure 1). This process allowed for multiple opportunities for feedback from representative RBS trained servers.

### Program specifications and aesthetic design

The first step in the process involved development of written program specifications that included the design of each program module, its functionality as a component within the website, the information architecture and instructional design that would best allow the content to engage the user and facilitate the learning and mastery of the material [10]. Program content was drawn from our experience with alcohol treatment programs as well as from the content required for RBS training programs in various states in the U.S., especially Oregon [11]. For example, the team determined how best to break the program content into *chunks* that worked best for Web delivery, ways that encouraged interaction and provided user with opportunities for self-evaluation.

#### Focus groups

Focus group procedures were informed by the team’s experience and by published guidelines [12]. These focus groups helped to identify the factors that would encourage participants to use the Web-based RBS program to achieve their work goals. Focus groups also provided early feedback about the type of content that needed to be included and how it would be best delivered.

Focus group sessions for the current project involved nine experienced alcohol servers, bartenders, and managers. Four of these participants were female and most were Caucasian, with one participant indicating more than one race. During the 1.5-hour sessions, focus group participants received a guided tour of the features of a preliminary or *alpha* version of the Web-RBS program. Participants were encouraged to discuss their feedback with the group regarding the overall "look and feel" of the program, its navigation, graphics, interactive features, and the amount, arrangement, and type of information presented. Participants were also asked about any information they felt was missing in the program and how the website compared to what they had experienced in the current workshop-based RBS training mandated by the Oregon Liquor Control Commission (OLCC). Sessions were audio taped and transcribed, which permitted us to identify themes and highlight possible changes to the program.

#### Usability testing

For optimal adoption and accurate use, Web-based tools like the RBS program should embody established usability standards [13,14]. A relatively small number of usability testers can provide extremely valuable data that inform revisions to a program [15,16]. The typical procedure calls for each user to meet individually with a research staff member who acts as a facilitator. During the session the usability tester is asked to interact with functional portions of the program using a *hands-on* approach as he/she accomplishes certain assigned tasks while receiving minimal direction from the facilitator. During this time each tester is asked to describe their thoughts using a *think-aloud* technique derived from cognitive science [17] that has proven effective in the study of human-computer interactions [18,19]. As noted by Hughes [20], the think-aloud technique provides “…direct, real-time observations [by] the user rather than self-reports such as surveys” (p.493). Think-aloud methods assess cognition concurrently with its occurrence, and thus may be better at describing the thoughts and attitudes of users rather than asking them for their retrospective self-reports [21]. At the conclusion of the session, testers were asked for their overall feedback including whether they would recommend the program to other servers, how interesting the program was, how much of the program information was new to them, whether important topics were omitted, how easy the program was to use, and how the program compared to in-person RBS training. Each usability test session was recorded and transcribed for review and analysis of themes and relevant statements.

Two rounds of usability tests were conducted. The first round involved seven alcohol servers and managers (six female and 1 male) of whom six described themselves as Caucasian, one as African American, and one as Hispanic/Latino (multiple categories were allowed). The second usability test consisted of 3 female alcohol servers and managers who described themselves as Caucasian and non-Hispanic/Latino. Usability testers were observed as they completed a series of tasks on the website including user registration, reviewing program information, completing section quizzes, and completing the online assessments.

### RBS program components

**Figure 2:**
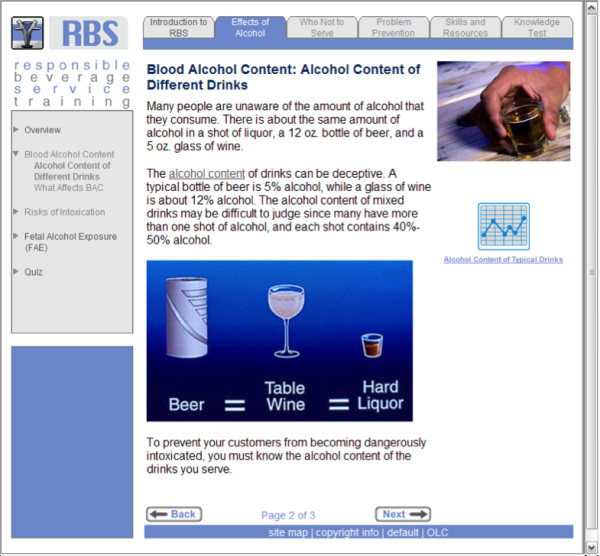
Example Webpage from Web-RBS Program.

The formative research steps described in this report directly informed the design of the resulting RBS Web-based training program for alcohol servers, managers and licensees. Other sources that shaped program design included the field experience of team members, feedback received from expert consultants (including RBS training directors in several states), and the requirements of the OLCC [11]. As depicted in Table 1, the Web-RBS intervention provided six modules for servers and another module for managers (see Table 1 and Figure 2).

**Table 1:** Components of Web-RBS program

**Introduction to RBS**	**Effects of Alcohol**	**Who Not to Serve**	**Problem Prevention**	**Skills and Resources**	**Knowledge Test**	**For Managers**
Alcohol safety and the customer	Alcohol content of different drinks	Carding procedures	Oregon DUII laws and liability for intoxicated driving	Carding (video)	Alcohol metabolism and development	State Systems for Regulating Alcohol
Alcohol safety and business	What affects blood alcohol content	Refusing service to underage customers	Preventing alcohol impairment	Refusing service to intoxicated customers (video)	Alcohol and car crashes and fatalities	RBS research
Alcohol safety and community	Alcoholism and problem drinking	Risks and legal issues of visible intoxication	Interactions between alcohol and other drugs	Preventing DUII and aggressive behavior (video)	Oregon alcohol laws and liability	House policies
Alcohol and safety and society	Alcohol and risk to minors	Recognizing visible intoxication	Preventing intoxicated driving	Preventing fetal alcohol exposure (video)	Effects of fetal alcohol exposure	Staff training
Oregon liquor license	Damage caused by fetal alcohol exposure	Preventing intoxication		Legal issues for alcohol service	Confidence handling underage customers, intoxicated patrons, and aggressive customers	Staff supervision
Oregon RBS program	Factors influencing risk in fetal alcohol exposure	Refusing service to intoxicated customers		Problem prevention	Confidence handling drinking drivers and pregnant women ordering alcohol	
	Typical effects of fetal alcohol exposure	Health impacts and legal issues of serving alcohol to pregnant women		Other resources	Satisfaction with establishment policies	
		Avoiding alcohol service to pregnant women			Satisfaction with the web-based training program	

The six separate server modules included: (1) Introduction to RBS training, (2) Effects of Alcohol, (3) Who Not to Serve, (4) Alcohol Problem Prevention, (5) Skills and Resources, and (6) an online Knowledge (certification) Test. Effective alcohol server responses to challenging situations were modeled in Modules 3–5 assisted by online role playing video vignettes. For example, videos demonstrated how to card (check the identification) of customers, ways to refuse service to an intoxicated customer, and how to discourage an intoxicated customer from driving. Each server module concluded with an interactive quiz that asked questions derived from the information covered in that module. A successful score on the Knowledge Test earned participants an informal certificate of program completion. The module for managers focused on their specific knowledge, roles, and responsibilities, including the State of Oregon system regulations, the *house* policies for risk reduction, self-protection, customer cooperation, training methods and the server-customer relationship, support and supervision through communications skills, and encouraging teamwork.

### Acceptability and feasibility study

We conducted a preliminary acceptability and feasibility study that assessed the extent to which participants using the RBS training program displayed improvements in their knowledge about alcohol service, attitudes about RBS service, self-efficacy, and intention to change their behavior in their establishments following the training experience. The Web-RBS program was made available to all staff who worked in an alcohol establishment, including servers, bartenders, and owners. Screening, informed consent, and study assessments were all completed online. Both Baseline and Post-test assessed alcohol server attitudes, knowledge, and self-efficacy. The Post-test also measured consumer satisfaction with the website.

#### Confidentiality of data management

All data associated with the study were maintained in a confidential manner. All transmission of data over the Internet was protected by our use of Secure Sockets Layer (SSL) – an industry standard encryption protocol. Study data were stored on secure servers located within a locked computer room that could only be accessed by approved study staff. In addition, each study participant was assigned a unique username and password which provided secure access to the program website. All study results were described only as aggregate data and never in a manner that could be used to identify any participant’s identity. Participants agreed to an online informed consent before they were able to interact with the Web-based intervention. The research protocol was approved by the Institutional Review Board of Oregon Research Institute.

### Recruitment

Study participants were recruited using informational announcements sent to local alcohol service establishments listed in the OLCC licensee database [22]. Establishments were considered eligible if they: (a) were located within any of three local Oregon counties (Linn, Benton, or Lane); (b) were independent establishments (not based in hotel, club, or larger organization); (c) had 12 or fewer alcohol service staff; and (d) had separate alcohol-serving and food service areas. The resulting sample of prospective service establishments that could be included in the study included rural, small town and small urban establishments.

An introductory recruitment meeting was held at each establishment attended by servers and establishment management. During this meeting, a research staff member provided an overview of the project. Topics included the study design, details about logging onto the RBS website to complete the screening, and the importance of completing the online informed consent. Printed materials were also left for any prospective participants who could not attend the meeting. Each study participant was given a card with a website address (url) and their unique registration information. Study participants had unique usernames and passwords, and they used this information to complete an online informed consent. Establishment owners/managers received $150 for supporting their staff to participate in the program. Study participants who completed Baseline and Post-test assessments received $50.

### Characteristics of establishments and participants

Twenty-two establishments with 112 participants were recruited for the feasibility and acceptability study of the Web-RBS intervention. More than 60% of participating establishments were tavern/bars. The typical participant was a 35 year-old female bartender or server with some college education, 10 years of hospitality service (7 years of alcohol service), and currently certified to sell alcoholic beverages in Oregon. More precise descriptions of participant characteristics are shown in Table 2.

**Table 2:** Participant characteristics at baseline

**Characteristic**		**Web-RBS**
		**(N = 112)**
Age (Mean yrs.)	34.10	(SD = 12.47)
Female (No.)	78	(69.6%)
Education (No.)		
No high school degree	3	(2.7%)
High School graduate	16	(14.4%)
Some college	61	(55.0%)
College graduate	30	(27.0%)
Post graduate	1	(0.9%)
Hospitality experience (Mean yrs.)	9.84	(SD = 9.42)
Alcohol service experience (Mean yrs.)	6.74	(SD = 7.95)
Length of RBS certification (Mean yrs.)	1.99	(SD = 1.49)

#### Measures

##### RBS knowledge

A set of 15 multiple choice questions was included on both the Baseline and Post-test assessments to measure knowledge of RBS content required by the State of Oregon and covered in our online RBS training program. For example, one item asked: "The brain is vulnerable to alcohol damage through the approximate age of 15, 20, or 25." Another item asked: "A server under 21 with a minor service permit in Oregon may: a) not mix drinks but may serve alcohol in a bar or lounge, b) only serve alcohol in areas where minors are permitted such as a restaurant, or c) serve alcohol from a kitchen but may not enter a restaurant bar or lounge)."

##### RBS attitudes and self-efficacy

The Baseline and Post-test assessments used a set of 40 items to test RBS attitudes related to content covered in our online RBS training. For example, one item asked respondents to rate the severity of the problem of fetal alcohol spectrum disorders and another item asked for a rating of confidence (self-efficacy) in dealing with types of challenging situations.

##### Consumer satisfaction

The Post-test included six Likert-scaled items that asked about whether the program provided new information useful for work, whether the information was personally interesting, whether they would recommend the program to other alcohol servers or owners/manager, how well organized the program was, and how they would compare the online RBS training program with live RBS training.

##### Program exposure

Unobtrusive measures of participant exposure to the RBS training were collected unobtrusively by the program, including the frequency and duration of each participant session and more detailed data on the viewing of individual program web pages [23].

### Statistical analyses

#### RBS knowledge

Each participant was assigned a score defined as the sum of knowledge items answered correctly (possible range = 0–15). Analysis of knowledge change from Baseline to Post-test was examined using paired *t*-tests. Individual item analysis was accomplished using classification table tests.

#### RBS attitudes and self-efficacy

The set of 40 attitude items was initially grouped into 7 subscales according to theme and similarity of rating scales. All items were scaled in a manner such that a higher rating reflected a more desirable or positive attitude. Subscales were further refined by examining their internal reliability (Cronbach's alpha; *a)* which resulted in dropping 2 scales thus yielding 5 subscales: Usefulness at Work (8 items; *a =* .65), Self-efficacy (8 items, *a =* .85), Risks (2 items, *a = *.66), Being Prepared (2 items, *a =* .87), and Establishment Policies (10 items, *a =* .92). A composite score for each subscale was assigned to each participant defined as the mean of ratings of subscale items. Baseline to Post-Test comparisons of attitudes used *t*-tests for independent samples for each subscale. Analysis of changes in attitudes from Baseline to Post-test was examined using paired *t*-tests.

#### Program exposure

Program exposure (use of the program) was the number of visits and overall time spent using the program [24].

## Results

A total of 84.8% (95/112) of study participants completed both the Baseline and the Post-test. The mean elapsed time between completion of the Baseline and the Post-test was 5.4 days (SD = 8.1, Median = 0, Range = 0–29).

### RBS knowledge, attitudes, and self-efficacy

#### RBS knowledge change (Baseline to Post-test)

Improvement in RBS knowledge was observed among 95 study participants by comparing the mean number of correct items out of the 15 item assessment at Baseline (Mean = 9.07, SD = 2.11) and the Post-test (Mean = 11.9, SD = 2.02). Paired *t*-test results revealed that this improvement was statistically significant (paired difference Mean = −2.82, SD = 2.44, t = −11.29, df = 94, p < .001). Analysis of individual items using classification tables tests revealed significant improvement on 73% (11/15) of knowledge items.

#### RBS attitude and self-efficacy (Baseline to Post-Test)

Scores of the five attitude subscales were examined using paired *t*-tests for change from Baseline and Post-test. In each instance, statistically significant improvements in mean scale ratings were observed (see Table 3).

**Table 3:** Attitudes, self-efficacy, and knowledge at Baseline and Post-test (N = 112)

**Measure**	**Baseline**		**Post-Test**		t*	**P**	***d***
	**Mean**	**(SD)**	**Mean**	**(SD)**			
Attitudes							
Usefulness †	0.45	(0.27)	0.72	(0.30)	−8.86	< .001	0.95
Risks	3.30	(1.54)	5.20	(0.99)	−11.78	< .001	1.47
Being prepared	5.08	(1.32)	5.73	(0.57)	−5.01	< .001	0.64
Establishment Policies	5.40	(0.81)	5.54	(0.73)	−2.47	.015	0.18
Self-efficacy	4.97	(1.08)	5.49	(0.71)	−5.10	< .001	0.57
Knowledge Change ‡	9.07	(2.11)	11.9	(2.02)	−11.29	< .001	1.29

* Paired *t*-test: 94 degrees of freedom and 2-tailed significance.

† Dichotomous items scored Yes = 1 and No = 0.

‡ Score defined as the sum of knowledge items answered correctly (possible range = 0–15).

### Consumer satisfaction

Almost all of the 93 study participants who provided consumer satisfaction data reported that they completed all sections of the program. Most reported that the program contained information that was new to them (31.2% indicated "a lot" and 59.1% indicated "some"), that it was useful for their work (41.9% indicated "a lot" and 48.4% indicated "some"), and that the information was personally interesting (32.3% indicated "a lot" and 53.8% indicated "some"). Similarly, 79.6% of participants reported that they found the program to be very well-organized and 82.8% reported that the program contained "about the right amount of information." They also reported that they would recommend the program to other alcohol servers or owners/managers (38.7% answering "very strongly recommend" and 39.8% "might recommend"). The majority of participants reported that they liked the online RBS program "much better" (32.3%) or "better" (26.9%) than live RBS trainings.

Similarly very positive results were obtained regarding participant ratings of usefulness of the 10 program sections (Table 4). Each of these sections was assigned a rating of “Very Useful” or “Useful” to around 80% of participants.

**Table 4:** Rating of the usefulness of Web-RBS program at Post-test (N = 93)*

**Feature, N (%)**	**Very useful**	**Useful**	**Only slightly useful**	**Useless**	**Did not visit**
Overview	35 (37.6%)	46 (49.5%)	12 (12.9%)		
Alcohol and minors	41 (44.1%)	44 (47.3%)	8 (8.6%)		
Intoxication	41 (44.1%)	42 (45.2%)	9 (9.7%)	1 (1.1%)	
Fetal Alcohol Spectrum Disorder	59 (63.4%)	30 (32.3%)	4 (4.3%)		
RBS research findings	39 (41.9%)	47 (49.5%)	7 (7.5%)	1 (1.1%)	
Hospitality industry viewpoint	36 (38.7%)	42 (45.2%)	12 (12.9%)	3 (3.2%)	
Legal information	50 (53.8%)	40 (24.0%)	3 (3.2%)		
Problem prevention ideas	44 (47.3%)	40 (43.0%)	7 (7.5%)	2 (2.2%)	
Section on Management	34 (36.6%)	41 (44.1%)	10 (10.8%)	2 (2.2%)	6 (6.5%)
Skills and Resources Section	34 (36.6%)	46 (49.5%)	9 (9.7%)	1 (1.1%)	3 (3.2%)

*Empty cells indicate no answers of that type were recorded.

### Program exposure

Most study participants visited the Web-based program on the first day it was made available for their use and participants visited an average 2.9 times (SD = 2.2; Median = 2.0). They viewed an average of 133.4 program webpages (SD = 50.5; Median = 148.5), and they spent an average of 113.2 min viewing the program (SD = 76.5; Median = 105.6).

## Discussion

The development of an innovative Web-based RBS training program was informed by formative research procedures and the contribution of a multidisciplinary design team. An acceptability and feasibility study of a completed version of this program was conducted with servers recruited from alcohol service establishments. Almost all participants completed the program, they improved their RBS knowledge, attitudes and self-efficacy, and they reported that they found the program to be useful, engaging, personally interesting and well-organized. The magnitude of these effects using Cohen’s d [25] was encouraging with three measures (attitude changes for usefulness and risks; knowledge change) showing a large effect size, two measures (attitude change on being prepared; self-efficacy change) showing a moderate effect size, and only one measure (attitude change on establishment policies) showing a small effect size. These overall strong positive findings were obtained despite the fact that participants had an average of 6.8 years (SD = 8.0) experience in alcohol service and an average of 9.8 years (SD = 9.4) experience in the hospitality industry. Greater changes in knowledge and attitudes would have been expected among novice servers. The present study is noteworthy because, to our knowledge, it represents the first reported empirical test of Web-based RBS training.

This study has some limitations. It is important to acknowledge that Baseline to Post-test changes may not indicate that participants had a deeper understanding of RBS content nor do they provide a test of long-term retention of relevant RBS content [26]. The present study did not assess possible changes in server on-the-job behaviors. A more controlled outcome study would include direct observation of pseudo patrons in establishment settings who would model the behaviors that a server would need to identify and respond to appropriately [27-33]. For example, these behaviors could likely include (1) carding of possibly underage patrons, (2) refusal to serve visibly intoxicated patrons, (3) preventing a visibly intoxicated patrons from driving, and (4) not serving visibly pregnant patrons.

It is also important to acknowledge that the efficacy of any brief RBS training intervention is likely to be enhanced were it to be used within the broader context of a community effort to institutionalize programs to prevent alcohol problems [34-36]. For example, Wallin and colleagues [36] have described how a concerted community-wide effort focusing on adoption, sustainability, key leader support, structural changes, and compliance resulted in the institutionalization of support for the prevention of alcohol problems in Stockholm, Sweden. This community support and compliance was associated with higher rates of refusing alcohol service to intoxicated and underage patrons and a significant reduction in violent crimes. Shults and colleagues [35] report similar positive results for multi-component programs (with RBS training) with community mobilization for reducing alcohol-impaired driving.

Finally, it remains for further research to examine other questions that could have important implications for RBS practices. For example, one research direction might examine the extent to which combining RBS classroom training with a Web-based program adjunct might prove more beneficial than providing training by either modality on its own. Additional research might also include direct behavioral interventions in alcohol service settings. Moreover, future research might examine the use of pseudo patrons and observational assessments with longer follow-up intervals to assess for meaningful longer-term improvements in RBS practices. It is clear from the significant number of people killed, injured, and affected by drunk driving in the United States that there is a public health need for ongoing research on evidence-based, effective RBS practices to reduce these staggering statistics. The current study has added to this body of research in an important way.

## Abbreviations

OLCC: Oregon Liquor Control Commission; RBS, Responsible Beverage Service: The appropriate sale and service of alcohol in commercial establishments.

## Competing interests

The authors declare that they have no competing interests.

## Authors’ contributions

BD participated in the design of the study, performed the statistical analysis, and helped draft the manuscript. JD conceived of the study and participated in the study design. TS participated in the study design, study coordination, and helped draft the manuscript. HS participated in the study design and helped draft the manuscript. MT participated in the study design, study coordination, and helped draft the manuscript. EM participated in study coordination and helped draft the manuscript. SC participated in the design and development of the website and the online evaluation of the intervention program. All authors read and approved the final manuscript.
